# FOXM1 and STAT3 interaction confers radioresistance in glioblastoma cells

**DOI:** 10.18632/oncotarget.12670

**Published:** 2016-10-14

**Authors:** Uday B. Maachani, Uma Shankavaram, Tamalee Kramp, Philip J. Tofilon, Kevin Camphausen, Anita T. Tandle

**Affiliations:** ^1^ Radiation Oncology Branch, National Cancer Institute, National Institutes of Health, Bethesda, Maryland, USA

**Keywords:** glioblastoma multiforme, FOXM1, STAT3, glioma stem cells, radio resistance

## Abstract

Glioblastoma multiforme (GBM) continues to be the most frequently diagnosed and lethal primary brain tumor. Adjuvant chemo-radiotherapy remains the standard of care following surgical resection. In this study, using reverse phase protein arrays (RPPAs), we assessed the biological effects of radiation on signaling pathways to identify potential radiosensitizing molecular targets. We identified subsets of proteins with clearly concordant/discordant behavior between irradiated and non-irradiated GBM cells *in vitro* and *in vivo*. Moreover, we observed high expression of Forkhead box protein M1 (FOXM1) in irradiated GBM cells both *in vitro* and *in vivo*. Recent evidence of FOXM1 as a master regulator of metastasis and its important role in maintaining neural, progenitor, and GBM stem cells, intrigued us to validate it as a radiosensitizing target. Here we show that FOXM1 inhibition radiosensitizes GBM cells by abrogating genes associated with cell cycle progression and DNA repair, suggesting its role in cellular response to radiation. Further, we demonstrate that radiation induced stimulation of FOXM1 expression is dependent on STAT3 activation. Co-immunoprecipitation and co-localization assays revealed physical interaction of FOXM1 with phosphorylated STAT3 under radiation treatment. In conclusion, we hypothesize that FOXM1 regulates radioresistance via STAT3 in GBM cells. We also, show GBM patients with high FOXM1 expression have poor prognosis. Collectively our observations might open novel opportunities for targeting FOXM1 for effective GBM therapy.

## INTRODUCTION

Glioblastoma multiforme or glioblastoma (GBM) continues to be the most frequently diagnosed and lethal of primary brain tumors. Radiotherapy remains a major approach to adjuvant therapy for patients with GBMs [1]. Extensive, diffuse parenchymal invasion is an important reason for failure of the most accepted treatment modalities, including surgical resection combined with radiation and chemotherapy [2]. It has long been recognized that tumors are heterogeneous in their radiation response and the degree of radiosensitivity was believed to be related to intrinsic properties (e.g., DNA repair capability and proliferation status) and to extrinsic properties of the tumor cell population [3]. The effects of radiation on malignant processes and the drivers of radio resistance have yet to be clarified. In the present study using reverse phase protein arrays (RPPAs) we assessed the biological effects of radiation on signaling pathways and demonstrate induction of Forkhead box protein M1 (FOXM1) with radiation treatment (RT). FOXM1 is a transcription factor and known to play an essential role in the regulation of a wide spectrum of biological processes, including cell proliferation, cell cycle progression, cell differentiation, DNA damage repair, tissue homeostasis, angiogenesis and apoptosis [4–7]. Recent evidence of FOXM1 as a master regulator of metastasis, over expression in human GBM and its important role in maintaining neural, progenitor, and GBM stem cells intrigued us to validate it as a radio sensitizing target [4,8,9]. Here we demonstrate that inhibition of FOXM1 radio sensitizes GBM cells. Further, we show that the radiation induced FOXM1 expression is dependent on STAT3 activation. Both FOXM1 and STAT3 proteins interact and co-localize in the nucleus under RT. We hypothesize that; these proteins (FOXM1/STAT3) together regulate radio resistance in GBM cells.

## RESULTS

### Proteomic profiling by reverse phase protein arrays (RPPA) identified induction of FOXM1 with RT

**Figure 1:**
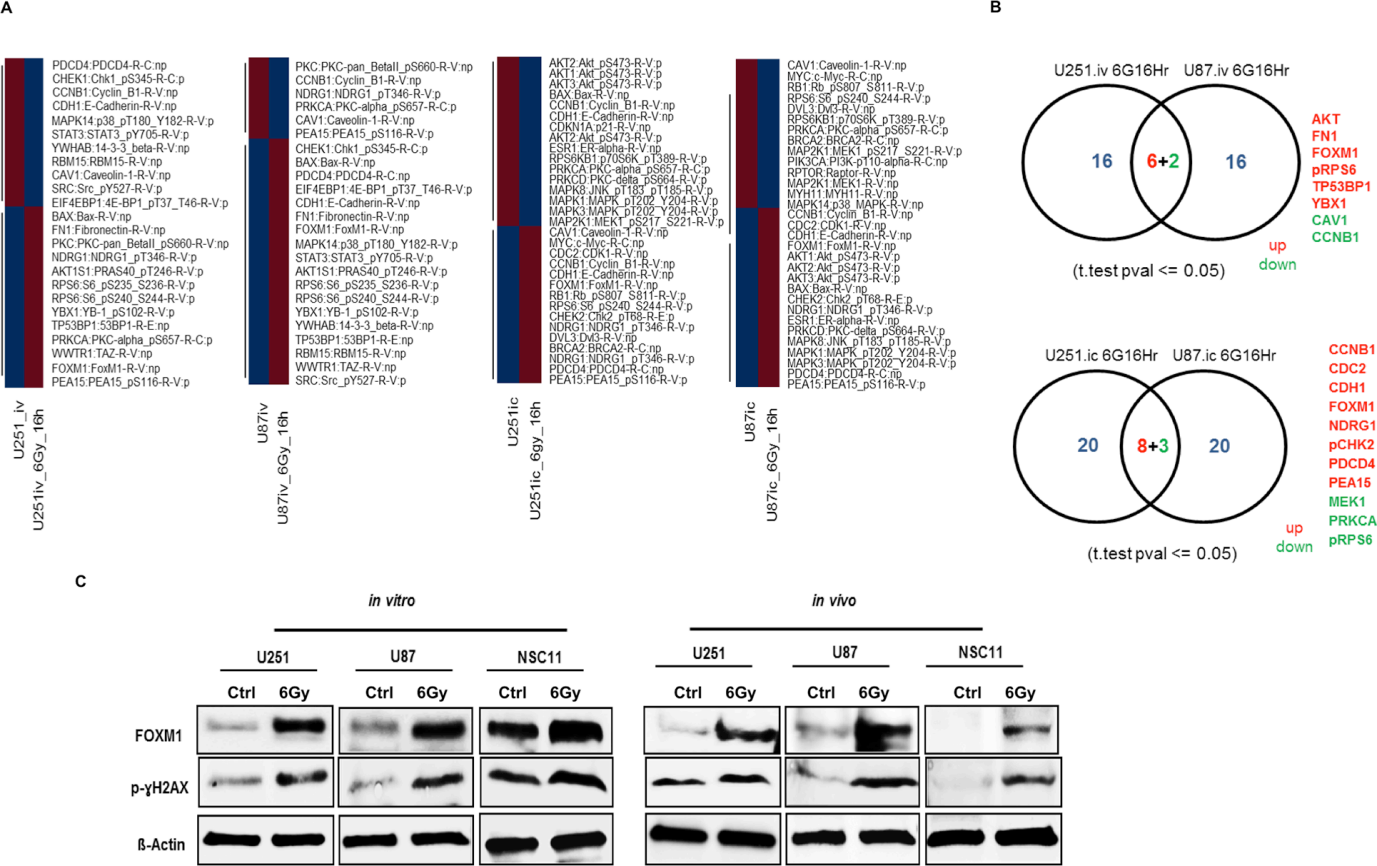
Proteomic profiling by reverse phase protein arrays (RPPA) identified induction of FOXM1 with RT Heatmap generated using correlation distance metric and hierarchical cluster analysis **A.** Protein intensity values are log2 and z-score transformed to remove any technical variation. Proteins changed by FC >1.2 (Red) FC < 1.2 (Blue) with reference to untreated samples were used for the analysis. Panel **B.** represents the venn diagram of commonly effected proteins between U251 and U87 cells. Radiation treatment (RT) induces increase in FOXM1 levels: panel **C.** represents the WB's for FOXM1 and p-γH2AX from lysates isolated for RPPA (see materials and methods for experimental and lysate preparation).

To determine the effects of radiation on signaling pathways in GBM, we assessed the modulation of phosphorylated and non-phosphorylated proteins using RPPA. Levels of 172 proteins were compared in U251 and U87 GBM tumor cells grown *in vitro* and *in-vivo* with and without RT. We identified subsets of proteins with differential expression between GBM cells grown *in vitro* and those grown *in vivo* in an orthotopic mouse model (Figure 1A). We observed upregulation of AKT, FN1, FOXM1, pRPS6, TP53BP1 and YBX1 and down regulation of CAV1 and CCNB1 in irradiated U251 and U87 cells grown *in vitro*. Under *in-vivo* conditions, we observed CCNB1, CDC2, CDH1, FOXM1, NDRG1, pCHK2, PDCD4 and PEA15 upregulation and MEK1, PRKCA and pRPS6 down regulation in irradiated U251 and U87 tumors (Figure 1B). However, FOXM1 was upregulated both *in vitro* and *in vivo* conditions after RT. Immunoblot analysis confirmed the increased levels of FOXM1 in irradiated GBM tumor cells (U251 and U87) (Figure 1C). We also observed RT induced upregulation of FOXM1 in the GBM stem cell line, NSC11 under both *in vitro* and *in vivo* conditions (Figure 1C).

### Genetic and pharmacologic FOXM1 inhibition affects GBM cell growth

**Figure 2:**
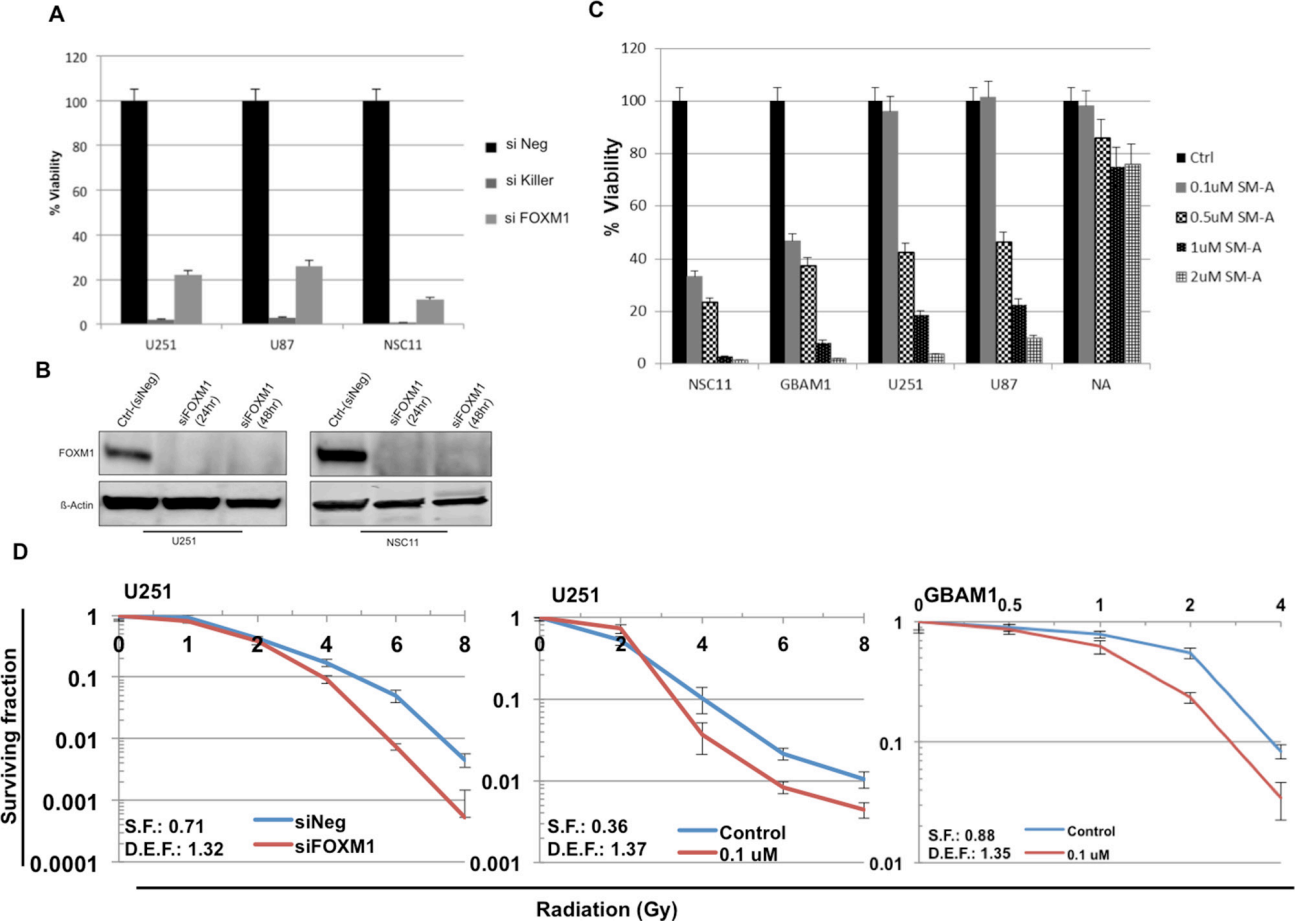
FOXM1 inhibition effects cell proliferation and sensitizes GBM cells to RT The human GBM U251, U87 and NSC11, cells transfected with siFOXM1, or negative (siNeg) siRNA in triplicate. Cell viability was assessed (Cell Titer Glow) at 96 hour after transfection **A. B.** western blot analysis of FOXM1 protein levels in siFOXM1 treated U251 and NSC11 cells. Panel **C.** represents bar graph for % cell viability in U251, U87, NSC11 and GBAM1 treated with Siomycin-A (0.1-2uM) or DMSO (control). Cell viability was assessed (Cell Titer Glow) 96 hour after treatment. Data is shown as Mean ± SD. Panel **D.** clonogenic survival assay in U251 and GBAM1 cells, with a dose enhancement factor (DEF) of 1.32 (siFOXM1) and 1.37 (0.1uM Siomycin-A) for U251 cells and DEF of 1.35 (0.1uM Siomycin-A) for GBAM1 cells. Values represent the Mean ± SD for three independent experiments.

Basal expression of FOXM1 was examined in various GBM stem cell lines and normal astrocytes. Seven out of eight GBM stem cell lines showed varied level of basal FOXM1 expression, whereas normal astrocytes did not express FOXM1 (Supplementary Figure S1A and S1B). Downregulation of FOXM1 by siRNA was also seen to inhibit GBM tumor cell and stem cell proliferation (Figure 2A). siNegative and siKiller were used as negative and positive controls respectively. siFOXM1 down regulated FOXM1 protein levels completely in two of the tested cell lines (U251 and NSC11) (Figure 2B). Using siomycin-A (SM-A), a small molecule inhibitor of FOXM1, we evaluated pharmacological inhibition of FOXM1 [10] and observed a concentration-dependent and statistically significant inhibition of cell proliferation in 5 different cell lines (Figure 2C). Except normal astrocytes, both GBM tumor (U87 and U251) and GBM stem cells (GBAM1 and NSC11) showed inhibition of cell proliferation. The results suggest that FOXM1 is required for growth of proliferating tumor cells but not for normal astrocytes (Figure 2C).

### FOXM1 inhibition sensitizes GBM cells to radiation treatment (RT)

Next, the effect of downregulation of FOXM1 on clonogenic survival of GBM tumor cells was examined. GBAM1 stem cells were selected as they harbor functional MGMT gene with resistance to standard GBM therapy (data not shown). Clonogenic survival analysis was done in U251 tumor cells and GBAM1 stem cells to measure the enhancement of radiosenstivity after FOXM1 inhibition. Cells were plated at specific clonogenic density, allowed to attach (6 hours), and treated with either siRNA (U251 cells) or siomycin-A (U251 and GBAM1 cells) 2 hours pre-irradiation. After RT, fresh drug-free medium was added, and colonies were stained 12 days later.

The survival efficiencies were 71% (U251 treated with siFOXM1), 36% and 88% (U251 and GBAM1 treated with SM-A respectively). Downregulation of FOXM1 resulted in an increase in the radiosensitivity of each of the two GBM (U251 and GBAM1) cell lines cell lines tested. The dose enhancement factors (DEF) at a surviving fraction of 0.1, was 1.32 for U251 treated with siFOXM1, 1.37 and 1.35 for U251 and GBAM1 treated with SM-A respectively. (Figure 2C).

### Effect of FOXM1 inhibition on repair of RT induced DNA double-strand breaks (DSB)

**Figure 3:**
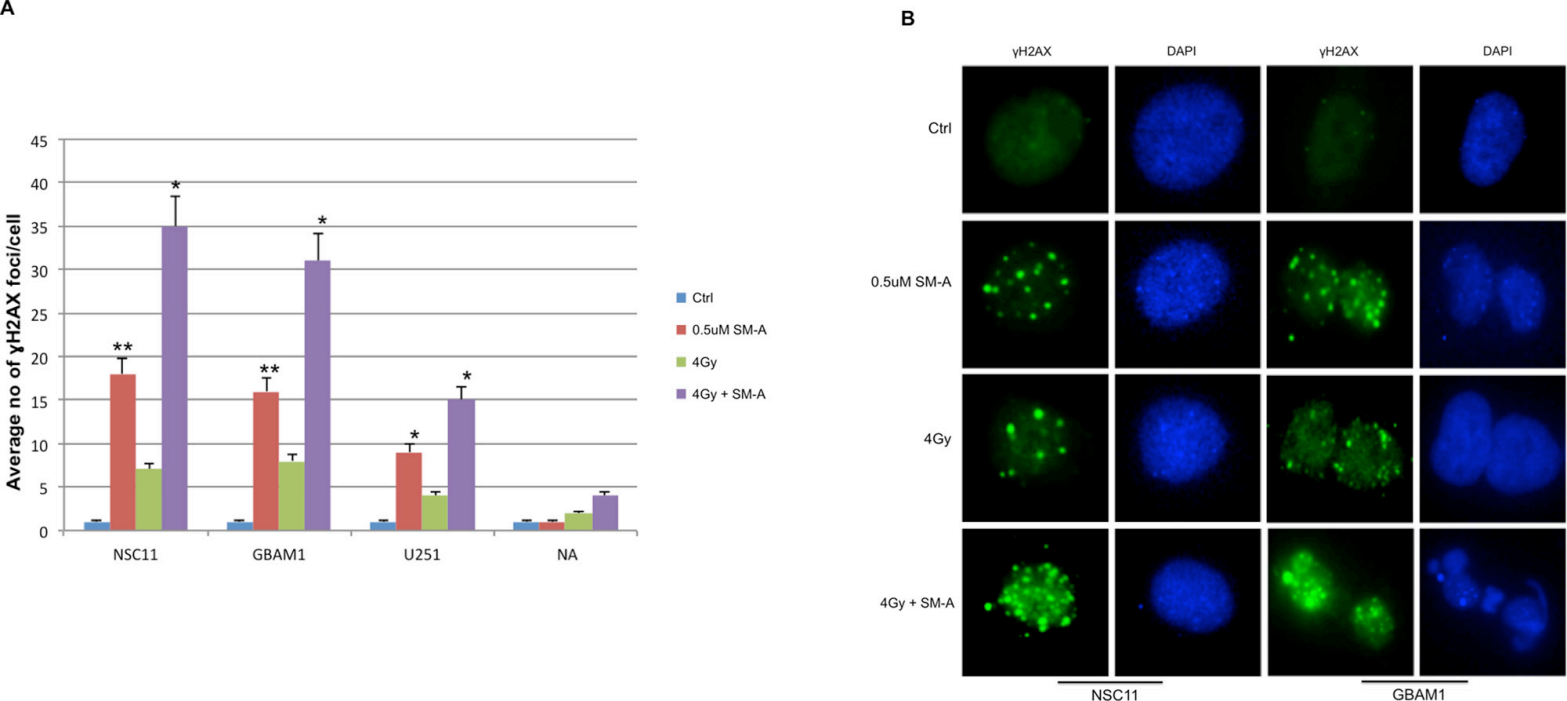
FOXM1 inhibition induces and enhances radiation induced DNA damage GBM cells (U251, NSC11 and GBAM1) and normal astrocytes (NA) were treated with Siomycin-A (0.5uM) or DMSO (control) with or without 4Gy IR. Cells plated in 4 well chamber slides were fixed 24hr after treatment and immunostained to detect γH2AX foci. Panle **A.** bar graph represents average no of γH2AX foci per cell. Foci were counted at 24 hours after treatment in at least 50 cells per experiment. Panel **B.** representative images of NSC11 and GBAM1 cells showing γH2AX foci, cells stained with anti-γH2AX antibody followed by Alexa Fluor 488–labeled secondary antibody (green) and nuclei were visualized with DAPI (blue) staining. Data presented is mean ± SD from three independent experiments. Student's t test was performed and the level of significance *indicate p < 0.05, **p < 0.005.

To assess the effects of FOXM1 inhibition on DNA damage and repair, RT induced double-strand breaks (DSB) were examined by γH2AX foci formation. Cells were treated with either SM-A alone or the combination of SM-A and radiation, and the average number of γH2AX foci at 24hr were counted. We observed significant (P<0.005) increased levels of γH2AX foci in SM-A plus RT GBM (NSC11, GBAM1, U251) cells, but not in normal astrocytes (Figure 3A). The results indicate persistence of RT-induced DNA-damage lesions after FOXM1 inhibition in GBM tumor stem cells, whereas the majority of DNA lesions were repaired in normal astrocytes. A significant (p<0.05) retention of γH2AX foci in NSC11 and GBAM1 cells treated with SM-A alone was also observed (Figure 3A and 3B). Representative images of γH2AX foci in NSC11 and GBAM1are shown (Figure 3B). These results suggest that inhibition of FOXM1 leads to incomplete repair of DNA double strand breaks in GBM tumor cells.

### FOXM1 inhibition with siomycin-A enhances RT induced mitotic catastrophe in GBM cells

**Figure 4:**
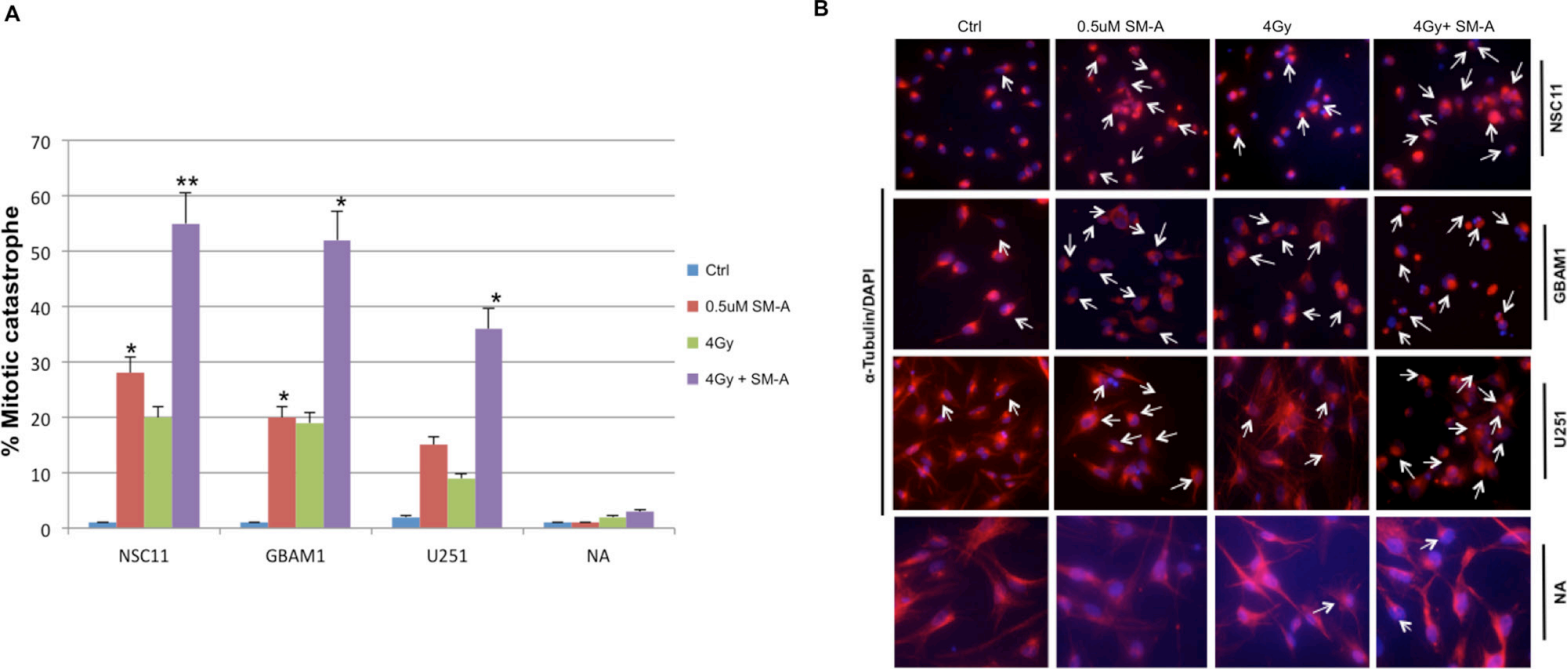
FOXM1 inhibition enhances radiation induced mitotic catastrophe Changes in nuclear morphology of U251, NSC11 and GBAM1 cells following 48hr exposure to Siomycin-A alone or in combination with 4Gy IR. Panel **A.**, quantitation of the percentage of multinucleated cells based on fluorescent microscopy analysis represented as bar graph. Panel **B.** representative pictures of cells stained with α-Tubulin and DAPI examined under a fluorescent microscope (magnification, 20X). Enlarged cells, containing multiple evenly stained nuclei (multinucleated cells) are characteristic for mitotic catastrophe (arrows). Data presented is the mean ± S.D, from three independent experiments. Student's t test was performed and the level of significance *indicate p < 0.05, **p < 0.005.

Due to FOXM1's known role in mitotic catastrophe and cell cycle progression [11, 12], mitotic catastrophe was measured in SM-A treated GBM cells. Cells undergoing mitotic catastrophe indicated by the presence of giant cells with multilobulated nuclei and aberrant mitoses, were visualized and scored by staining with anti-tubulin antibody (red) and nuclei with (blue) at 48hr after SM-A treatment (Figure 4). We observed a significant increase (p <0.05) in percentage of GBM cells undergoing mitotic catastrophe after concurrent SM-A and RT treatment (p < 0.005 NSC11 & GBAM1, p <0.05 U251), whereas normal astrocytes showed minimal mitotic catastrophe (Figure 4A and 4B). These results indicate that one of the mechanisms of cell death is the induction of mitotic catastrophe.

### FOXM1 regulates genes associated with cell cycle progression, DNA repair and its inhibition effects homologous recombination (HR) DNA repair

**Figure 5:**
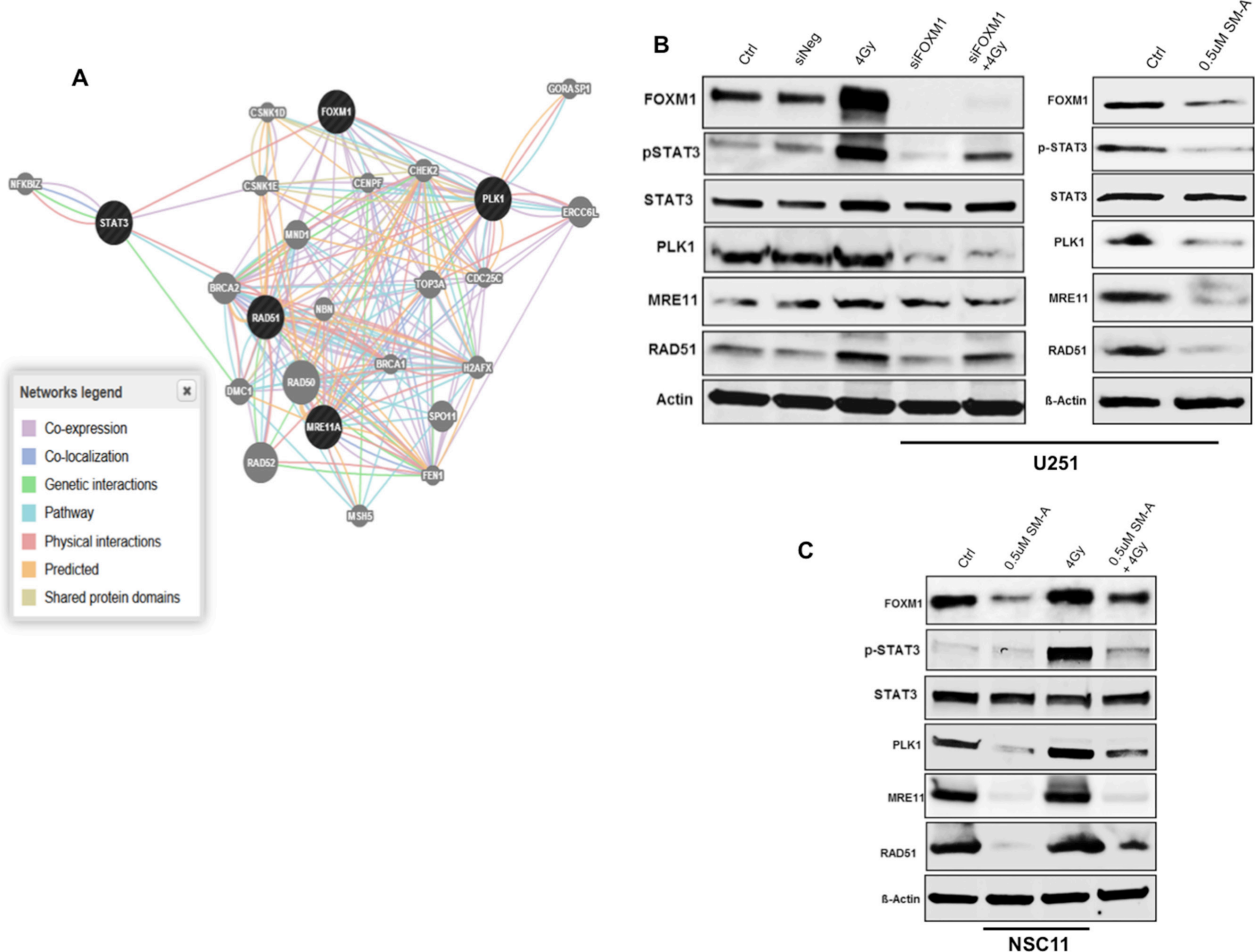
FOXM1 regulates genes associated with cell cycle progression and DNA repair Panel **A.** shows the molecular functional network map of canonical pathways including coexpression, physical interaction, and predicted networks of FOXM1 analyzed by GeneMANIA (http://genemania.org/) tool. Panel **B.** represent immunoblots of lysates prepared from U251 cells (panel **C.** NSC11 cells) treated (24hr after treatment) as indicated for molecules involved in DNA repair (MRE11, RAD51), chromosomal segregation (PLK1) and cell survival (total STAT3 and phospho STAT3). A representative data from three independent experiments is shown.

To characterize the molecular mechanisms underlying the enhanced radio sensitivity after FOXM1 inhibition, the online GeneMANIA tool (http://www.genemania.org/) was used to build a predictive molecular functional network map of canonical pathways and the associated genes with FOXM1 [13]. The networks associated with FOXM1 involved, DNA repair, chromosomal segregation and cellular survival. Specific molecules from these pathways and their complex interactions are shown (Figure 5A). Several of these molecules have been previously reported to have interactions with FOXM1. We confirmed their expression in GBM cells treated with either siFOXM1 or SM-A by immunoblot analysis (Figure 5B and 5C). We observed decreased expression levels of genes associated with DNA repair (MRE11, RAD51) and genes involved in cell cycle and chromosomal segregation (PLK1) after FOXM1 inhibition in both GBM tumor cells and stem cells. Although we observed lower levels of MRE11 in cells treated with SM-A but not in cells treated with siFOXM1 (Figure 5B and 5C). We attribute this difference to wider effects of pharmacologic inhibition compared to more specific siRNA effects.

**Figure 6:**
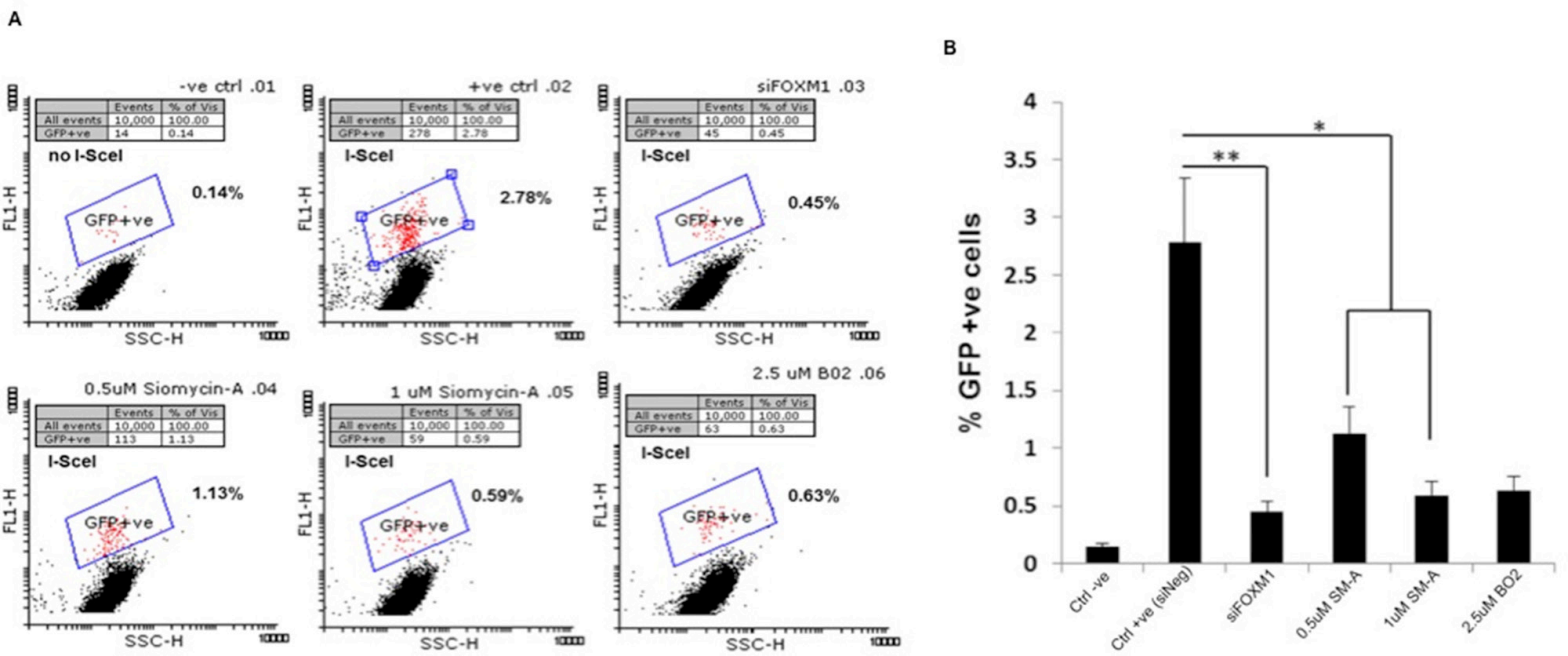
FOXM1 inhibition effects Homologous recombination (HR) DNA repair To assay homologous recombination (HR), U251 DR-GFP cells were transfected with pCBAScel vector 24 hours prior to indicated treatments. GFP+ve cells representing HR repair events were determined by flow cytometry **A.** represents two-dimensional plot of GFP-specific fluorescence –FL1 (on Y axis versus light scatter (x-axis) to identify GFP+ve cells, **B.** represent bar graph of percentage GFP+ve cells. Two different concentrations of BO2 (RAD51 inhibitor) were used as a positive control. Data presented are the mean ± SD from at least three independent experiments. Student's t test was performed and the level of significance *indicate p < 0.05, **p < 0.005.

Given this altered expression in key members of the DNA damage response MRE11 and RAD51, we next investigated whether FOXM1 affects DNA repair efficacy, by examining one of the major DNA-double strand break (DSB) repair pathway, homologous recombination (HR) pathway. We examined HR in U251 cells using DR-GFP plasmid as described (Supplementary Methods). The HR assay relies on the two inactivated tandem repeat (DR-GFP) transfected cells to express GFP detected by flow cytometry. U251 cells transfected with siNegative was used as a negative control and U251 cells transfected with siNegative and DR-GFP plasmids as a positive control (Figure 6). Positive control cells showed 2.78% GFP positive cells. Cells transfected with either siFOXM1 (p<0.005) or treated with SM-A (p<0.05) showed significant decrease in percentage GFP+ cells compared to negative control cells (Figure 6). These results indicate FOXM1 abrogation affects DNA-DSB repair efficacy by inhibiting HR-DSB repair, further validating FOXM1's role in DNA damage and repair response.

### Radiation induced activation of STAT3 and FOXM1 induction is mutually co-regulated; FOXM1/STAT3 interacts and co-localize following RT

**Figure 7:**
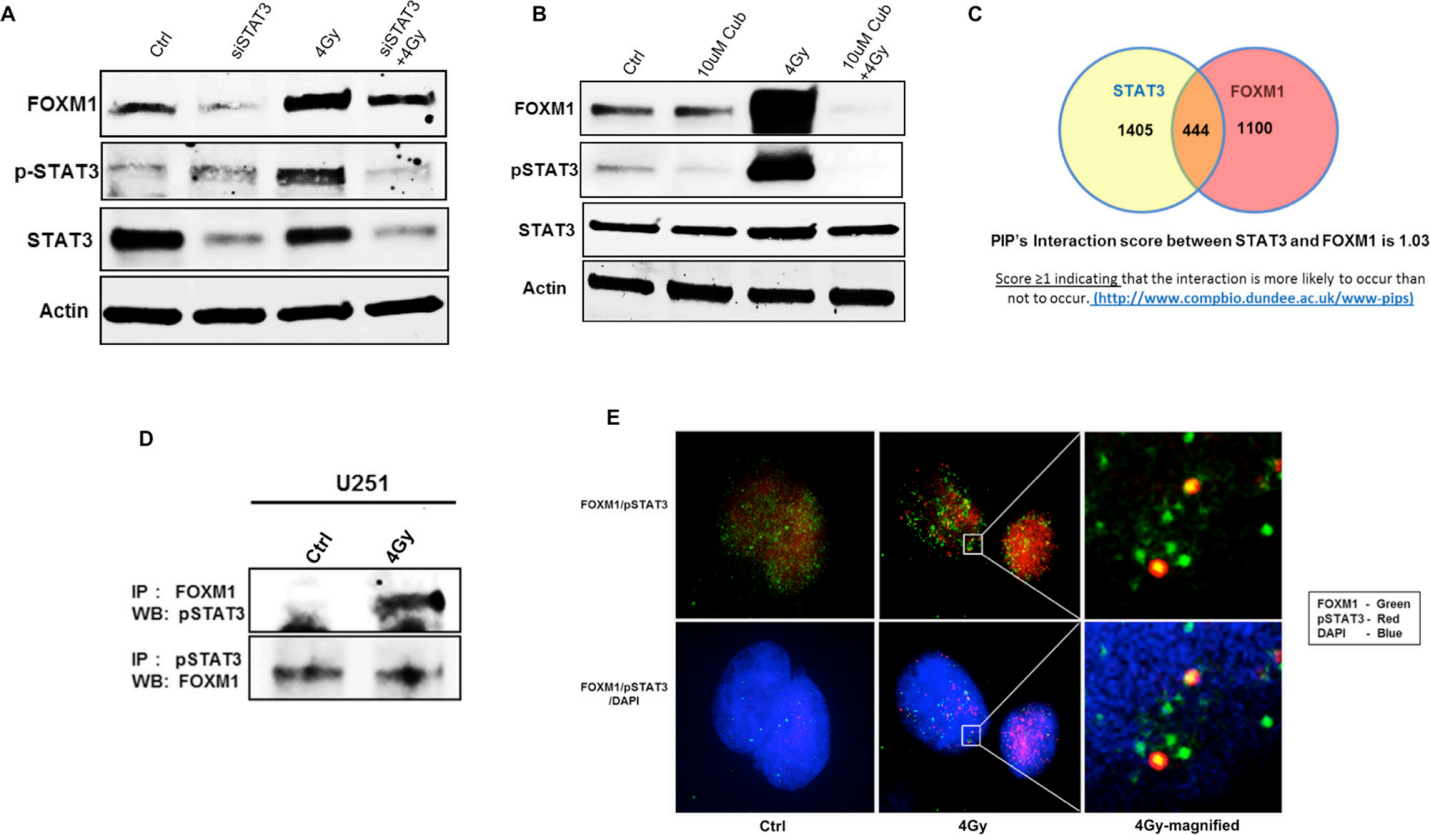
FOXM1 expression and STAT3 activation are mutually co-regulated under RT Panels **A, B.** represent immunoblots (for FOXM1, total STAT3, phopho STAT3) of lysates prepared from siRNA mediated inhibition of STAT3 (A) and STAT3 inhibitor (Cucurbutacin-I) (B) as indicated. Panel **C.** represents the Venn diagram of no of interacting molecules among and between FOXM1 and STAT3 along with their predicted PIP's interaction score. Co-immunoprecipitation immunoblots are represented in panel **D.** for FOXM1 and pSTAT3 interaction and co-localization in the nucleus represented by Immunofluorescence images in panel **E.** FOXM1(green), pSTAT3(red), nuclear stain -(blue). Data presented is representative of three independent experiments.

Based on the interaction map (Figure 5A) and recent evidence showing the FOXM1 is a STAT3 transcriptional factor target [19], we assessed an association between the two molecules. To downregulate STAT3 in U251 cells we used either siSTAT3 or Cucurbutacin-I, a STAT3 inhibitor (Figure 7). We observed radiation induced increase in phosphorylated STAT3 (pSTAT3) levels in U251 cells (Figure 7). However, RT-induced pSTAT3 levels and total STAT3 levels were decreased in cells with downregulated STAT3 (Figure 7A). We also observed lower levels of pSTAT3 in Cucurbutacin-I treated U251 cells (Figure 7B). Moreover, STAT3 downregulation also decreased RT induced FOXM1 levels in U251 cells, suggesting an association between FOXM1 and STAT3 expression. Thus, to comprehend the interaction between FOXM1 and STAT3, we performed a predictive human protein-protein interactions (PIP's) (http://www.compbio.dundee.ac.uk/www-pips) [20]. PIPs predicted a score of 1.03 between STAT3 and FOXM1 interaction, a score ≥1 indicating that the interaction is likely to occur (Table 1). STAT3 has 1405 interactors and FOXM1 has 1100 interactors, of which there are 444 common interactors (Figure 7C). Further to see if there is any physical interaction between the two proteins, we used immunoprecipitation (IP) assay technique. Immunoprecipitation reaction was carried out using anti-FOXM1 as a precipitating antibody followed by immunoblotting using anti-STAT3 and vice-versa (Figure 7C). On IP assay, pSTAT3 was co-imunoprecipitated with RT-induced FOXM1 (Figure 7D, upper panel). However, we did not see FOXM1 immunoprecipitation with pSTAT3 antibody (Figure 7D, lower panel). So we confirmed the physical association between these two proteins using two more approaches. We performed IP reactions with STAT5 antibody, another member of STAT family. The PIP score of 0.049 between FOXM1 and STAT5 did not indicate any association between FOXM1 and STAT5 (Table 1). We verified this observation of no interaction between these two proteins on an IP assay (Supplementary Figure S1C). Next, we confirmed co-localization between FOXM1 and STAT3 using an immunofluorescence imaging (Figure 7E). We observed an increase in co-localization of FOXM1 and STAT3 in the nucleus of RT treated U251 cells (Figure 7E). Collectively, these results suggest a co-regulatory positive feedback loop mechanism between FOXM1 expression and STAT3 activation.

**Table 1:** Evidence for Interaction between FOXM1 and STAT3

Common Interactor	Name of Common Interactor	Score for STAT3-Interactor	Score for FOXM1-Interactor
STAT6	STAT6: Signal transducer and activator of transcription 6	22.50	0.049
STAT4	STAT4: Signal transducer and activator of transcription 4	14.80	0.083
STAT1	STAT1: Signal transducer and activator of transcription 1-alpha/beta	14.80	0.083
STAT5A	STAT5A, STAT5: Signal transducer and activator of transcription 5A	12.60	0.049
STAT5B	STAT5B: Signal transducer and activator of transcription 5B	8.34	0.083
STAT2	STAT2: Signal transducer and activator of transcription 2	0.109	0.083
STAT3	STAT3: Signal transducer and activator of transcription 3	-	1.03

Table 1 shows PIP's Interaction score between FOXM1 and STAT3 with other STAT molecules. The PIPs database (http://www.compbio.dundee.ac.uk/www-pips) is a resource for studying protein-protein interactions in human. It uses a naïve Bayesian classifier to calculate probability of interaction between two proteins combining Gene Co-expression, Orthology, Domain Co-occurrence, Co-localization, Post Translational Modification, Network Analysis. Score ≥1 indicates that the interaction is more likely to occur than not to occur.

### High FOXM1 expression is associated with decreased survival in patients with GBM and other cancer histologies

We next evaluated the prognostic effect of FOXM1 expression on patient survival using univiariate Kaplan-Meier plots, by curating publically available datasets; REMBRANDT (http://rembrandt.nci.nih.gov), for gliomas (Supplementary Figure S2A). Low FOXM1 expression (128 patients) was associated with better survival (p=7.26E-9) when compared to high FOXM1 expression (263 patients). We found FOXM1 expression to be a significant marker of prognosis in GBM. We also compared FOXM1 expression to survival analysis in breast and lung cancer patients using KM-Plotter (www.kmplot.com) [21, 22]. Kaplan-Meier curve analysis showed patients with FOXM1 expression above the median had significantly poorer survival compared to those with FOXM1 expression below the median, both in breast cancer (1115 patients, p=2.7e-07) (Supplementary Figure S2B) and lung cancer (1210 patients, p=3.2e-11) (Supplementary Figure S2C). The survival analysis shows that individuals with tumors expressing low levels of FOXM1 had a significant survival advantage when compared with individuals with tumors expressing high levels of FOXM1, not only in GBM patients but also in other cancer histologies. Our results indicate FOXM1 expression as a potential prognostic marker as well as a putative molecular target for cancer therapy.

## DISCUSSION

Glioblastoma multiforme is the leading cause of death in adult patients with primary brain tumors and remains one of the most lethal cancers. Though radiotherapy remains the standard of care, local recurrence after treatment continues to pose major hindrance to this type of therapy. Growing evidence suggests that there is a distinct population of cancer cells with exclusive capability to self-renew, termed glioma stem cells (GSCs), which are responsible for cancer initiation, propagation, and maintenance [23–26]. Studies have suggested that the resistance of GSCs to current therapies is, related to current treatment failures. [2]. Thus, better understanding of molecular changes that cause radio resistance in these cells will help to design therapies targeting GSCs and to prevent possible recurrence.

In this present study using reverse phase protein arrays (RPPAs) [27], we assessed the biological effects of radiation on signaling pathways to identify potential radio sensitizing molecular targets and identified FOXM1 as a potential radio sensitizing molecule. The potential for FOXM1 inhibition in glioma treatment has been suggested previously [28, 29]. However, this is the first study to demonstrate that FOXM1 inhibition causes radiosensitization in GBM cells by modulating DNA repair.

We observed varied basal expression of FOXM1 in different patient derived GBM stem cells and its induction with RT. FOXM1 is known to be overexpressed in various human malignancies [30] and its expression in GBM correlates with the tumorigenicity [31]. FOXM1's overexpression in various tumors including GBM [8,9] suggests a role in cell proliferation [4]. Recently a thiazole antibiotic SM-A, was identified with a high-throughput cell-based screen as an inhibitor of the transcription factor FOXM1. SM-A inhibits the transcriptional activity and the expression of FOXM1 [10], but the exact mechanism is not clear. *Gartel et al.*, proposed SM-A acts by effecting proteasomal degradation of a negative regulator of FOXM1 (NRFM), which in turn inhibits the activity of FOXM1 as a transcription factor [32, 33]. We show in this study that inhibition of FOXM1 (siRNA or SM-A) significantly affects viability and radiosensitizes GBM cells. FOXM1 expression is restricted to cells that are proliferating and is negatively regulated in quiescent or terminally differentiated cells [9, 34]. Which is corroborated by our observation of negligible levels of FOXM1 and no significant effect with its inhibition on normal astrocytes.

Moreover, FOXM1 inhibition induced DNA damage, decreased DNA repair and increased mitotic catastrophe, which were further enhanced when combined with RT, suggests its role in DNA damage pathways. FOXM1 involvement in DNA damage pathways, has been shown previously where FOXM1-deficient mouse embryonic fibroblast cells showed increased levels of DNA damage [35]. As FOXM1 plays a role in cell proliferation, this induction of DNA damage and mitotic catastrophe may be related to the necessity of functional FOXM1 for proper mitotic progression and to ensure genomic stability [7,11,12]. Since chromosome segregation errors result in structural chromosome aberrations leading to DSBs [36], our results showing increased levels of γH2AX foci (DNA damage marker), in FOXM1 inhibited GBM stem cells lend further support to FOXM1 role in maintaining genomic stability.

We also observed decreased levels of MRE11 and RAD51, proteins that are associated with DNA repair. Our results are consistent with reports describing RAD51 and MRE11 as transcriptional targets of FOXM1 [14, 15]. A recent study has shown that FOXM1 is required for DNA double strand break (DSB) repair by homologous recombination (HR) but dispensable for non-homologous end-joining (NHEJ) repair [5]. Glioma stem cells (GSCs) are known to contribute radioresistance traits through preferential activation of the DNA damage checkpoint response and increased DNA repair capacity [37]. Our observation of decreased homologous recombination (HR) repair activity in FOXM1 inhibited cells suggests its role in DSB-DNA repair, making it an attractive sensitizing target for radiation which render cells dependent on DNA repair mechanisms.

FOXM1 is also known to play important role in cell cycle progression and stimulate expression of a number of genes that are critical for the G2 –M progression; such as Plk1, Aurora B, Cyclin B1, CDC25B, CENP-A, and Survivin [11]. Consistent with these reports, we observed decreased levels of PLK1, in FOXM1 inhibited GBM cells. This decreased expression of a cell cycle progression gene may be attributed to the induction of mitotic catastrophe in GBM stem cells with an enhanced effect with RT. Irradiation is known to prompt the phosphorylation of STAT3 leading to nuclear localization [38] and modulate transcription of a variety of genes involved in the regulation of critical functions, including cell differentiation, proliferation, apoptosis, angiogenesis, and metastasis [16, 17, 39]. The STAT3 signaling pathway is also implicated in resistance to radiation in GBM CSCs [18]. Further, recent evidence showing the FOXM1 gene as a new STAT3 transcriptional factor target and its transcriptional dependency on STAT3 signaling activation [19] suggests a direct or indirect co-regulatory mechanism between FOXM1 and STAT3 activation. Co-immunoprecipitation and immunofluorescence studies show physical interaction and co-localization of FOXM1 with STAT3. Inhibiting STAT3 expression or activation repressed FOXM1 expression, indicating dependency of FOXM1 expression on STAT3. We also demonstrated FOXM1's role in STAT3 activation with RT suggesting a positive feedback loop mechanism between FOXM1 expression and STAT3 activation. Recent evidence showing FOXM1 regulates STAT3 activation and STAT3 expression [40] strengthens this idea, though we did not observe a significant decrease in total STAT3 protein under FOXM1 inhibition. More studies are needed to understand the exact mechanism of this interaction (FOXM1/STAT3) and their co-regulation. Given that FOXM1 is involved in a positive feedback loop and activates its own transcription [41], we hypothesize FOXM1/STAT3 interaction together drives FOXM1 expression and might regulate resistance to radiation in GBM stem cells (Supplementary Figure S3). Also, our univariate survival analysis showed that low expression of FOXM1 had a significant survival advantage over individuals with tumors expressing high levels of FOXM1 in GBM, breast and Lung cancer patients. These findings suggest FOXM1 utility as a prognostic marker and a potential molecular target not only for GBM therapy, but also for other cancer histologies.

GSCs are intrinsically resistant to conventional therapies, particularly radiation, and are implicated in radioresistance. Here we show targeting FOXM1 leads to radio sensitization of GSCs. Evidence of FOXM1 having a significant role in DNA damage response, cancer drug resistance and its overexpression associated with GBM tumorigenicity, make it a promising and potential radiosensitizing target for GBM therapy. FOXM1 could also be an important biomarker for identifying patients who respond better to current GBM treatment modalities.

## MATERIALS AND METHODS

### Cell lines & Drugs

U25, U87 (National Cancer Institute Frederick Tumor Repository) human GBM cell lines were grown in Dulbecco's Modified Eagle Medium (DMEM) (Invitrogen, Carlsbad, CA) with 10% fetal bovine serum (FBS), and maintained at 37°C, 5% CO2. The neurosphere forming cultures NSC11 (kindly provided by Dr. Frederick Lang, M. D. Anderson Cancer Center) and GBAM1 stem-like cells, were established from patient resections, grown as previously described [42–44]. Neurospheres were maintained in stem cell medium (Dulbecco's modified Eagle's medium—Ham's F-12 Nutrient Mixture (DMEM-F-12) supplemented with B27 Supplement (Life Technologies), 20 ng/ml epidermal growth factor, 10 ng/ml basic fibroblast growth factor (Sigma-Aldrich)) and maintained at 37°C in an atmosphere of 5% CO2. Human brain astrocytes (normal astrocytes) were purchased from ScienCell (#1800, Carlsbad, CA) and grown in Astrocyte Medium with the recommended supplements as per manufacturer's instructions and used between passages 3-6. Siomycin-A an antibiotic that inhibits FOXM1 was obtained from Millipore, MA (#567060) and NCI-Chemotherapeutic Agents Repository., USA.

### RPPA analysis

Tumor lysates were prepared from GBM tumor cells (U251and U87) and U251 tumors (N=3) grown orthotopically in nude mice. The lysates were prepared in RPPA lysis buffer [1% Triton X-100, 50 nmol/L Hepes (pH 7.4), 150 nmol/L NaCl, 1.5 nmol/L MgCl2, 1 mmol/L EGTA, 100 nmol/L NaF, 10 nmol/L NaPPi, 10% glycerol, 1 nmol/L phenylmethylsulfonyl fluoride, 1 nmol/L Na3VO4, and aprotinin 10 μg/mL. The RPPA analysis was carried out by RPPA Core Facility, MD Anderson Cancer Center, Houston, Texas. Briefly, 5 serial dilutions of lysates were arrayed on nitrocellulose-coated slides, probed with (172 phosphorylated and phosphorylated) antibodies, and visualized by DAB colorimetric reaction [27]. Relative protein levels for each sample were determined by interpolation of each dilution curves from the standard curve antibody slide. All the data points were normalized for protein loading and transformed to a linear value. Linear values were transformed to Log2 value and then median-centered for hierarchical cluster analysis. The Heatmap was generated using correlation distance metric and hierarchical cluster analysis. Protein intensity values are log2 and z-score transformed to remove any technical variation. Proteins changed by FC >1.2 (Red) FC < 1.2 (Blue) with reference to untreated samples were used for the analysis. The RPPA data used in this analysis can be found at http://www.ncbi.nlm.nih.gov/geo/ (GSE70776).

### FOXM1 inhibition and cell viability

Siomycin A, an antibiotic thiazole compound (FOXM1 inhibitor) (cat no: sc-202339, Santa Cruz., USA) was reconstituted in dimethyl sulfoxide (DMSO) and stored at −20 C. Cells were plated overnight prior to drug treatment and treated at concentrations indicated in each experiment. Cell viability was assessed five days post drug treatment through quantification of ATP levels (CellTiter-Glo luminescent Reagent, Promega, Madison, WI). For siRNA mediated FOXM1 inhibition, 2-pmol siFOXM1 (FlexiTube GeneSolution GS2305 for FOXM1 (contains 4 validated siRNAs for FOXM1) (Qiagen Inc., Germantown, MD) was complexed with RNAi Max lipid transfection reagent (Invitrogen) in DMEM media for 15 minutes at room temperature. Cells suspended in DMEM supplemented with 20% FBS were then added. Plates were maintained at room temperature for 15 minutes before being placed at 37 C/5% CO2. Negative (All star siNegative [siNeg], Qiagen) and positive (All star siCelldeath, Qiagen) control siRNAs were used as controls. 48 hours post transfection cells were processed as indicated.

### Western blot analysis

Cell pellets were lysed on ice in RIPA buffer (Pierce, Rockford, IL) supplemented with Complete Mini EDTA-free Protease Inhibitor Cocktail (Roche, Indianapolis, IN) and Phosphatase Inhibitor Cocktail (Sigma, St. Louis, MO). Protein concentrations were determined by Bradford assay (Bio-Rad, Hercules, CA). Protein(50ug) was diluted 1:5 in 5X protein loading buffer (Fermentas, Glen Burnie, MD), boiled at 80°C for 5 minutes, electrophoresed on a 4-20% Tris-Glycine gel, and transferred using a Trans-Blot Turbo Transfer System (Bio-Rad, Hercules, CA). Membranes were blocked in 5% Non-fat milk powder (BioRad), incubated with primary antibody overnight at 4°C, incubated with HRP-coupled secondary antibody 1 hour at room temperature, developed with Visualizer Western Blot Detection Kit (Millipore, Billerica, MA), and visualized on a LAS-4000 imager (Fujifilm, Edison, NJ). The following antibodies were used at 1:1000 dilutions: Rabbit anti-FOXM1 (#5436), PLK1(#4513), pCDC2(#9111), CDC2 (#9112), MRE11, Survivin (#4895), mouse anti-STAT3(#9139), pSTAT3 (#9138), ß-Actin (#3700) (Cell Signaling Technology., MA); mouse-anti -pγH2AX (#05636, Millipore., MA), rabbit-anti RAD51 (sc-8348, Santacruz., CA), mouse anti-53BP1 (#612522, BD Transduction Laboratories., CA). Secondary antibodies, goat anti-rabbit-HRP, goat anti-mouse-HRP (Santa Cruz, CA) were used at 1: 10,000 dilution.

### Immunofluorescence and staining for γH2AX

Immunofluorescence staining and counting of γH2AX nuclear foci was performed as previously described [45]. Slides were mounted and images captured using Olympus FSX100 fluorescent microscope. For each treatment condition, foci were determined in at least 150 cells. Image-J (NIH) software was used to analyze the mean number of foci with combined area of γH2AX foci per nucleus.

### Mitotic catastrophe

Immunofluorescent staining and counting of mitotic catastrophe was performed as described [46]. NSC11 Cells were seeded in four-well chamber slides, after indicated treatments, 10% serum were used to make the cells adherent. Cells were fixed with methanol for 15 minutes at −20°C, washed three times with PBS, blocked with 1% BSA (in PBS) three times for 10 minutes, and stained overnight at 4°C with mouse anti-+-tubulin antibody (Sigma) at 1:500 dilution. Cells were washed three times with 1% BSA, and were stained with goat anti-mouse- Alexa Fluor 594 (Invitrogen) at 1:500 dilution for two hours at room temperature. Cells were washed three times with 1% BSA and slides were mounted in Vectashield mounting medium with DAPI (Vector Labs, Burlingame, California). Images were viewed and captured on a fluorescent microscope (Olympus FSX100 microscope). The presence of giant cells with multi-lobulated nuclei and aberrant mitoses was the criterion for defining cells undergoing mitotic catastrophe. For each treatment condition 150 cells were scored; the average of three separate counts of the same cells is reported.

### Clonogenic survival assay

Clonogenic survival assay was performed as described elsewhere [46]. Established U251 GBM cells and GBAM1 cells were seeded into six-well tissue culture plates and allowed to attach for six hours. For combination treatment, siomycin-A or DMSO control was added to the culture media for 4 hours followed by RT and change of fresh media. Twelve days after seeding, colonies were stained with crystal violet. The number of colonies containing at least 50 cells was determined and the surviving fractions were calculated. For combination treatment, survival curves were generated after normalizing for the cytotoxicity generated by siomycin-A alone.

### Functional network map and survival analysis

Online GeneMANIA tool [GeneMANIA (http://www.genemania.org) is a flexible, user-friendly web interface for generating hypotheses about gene function, analyzing gene lists and prioritizing genes for functional assays] was used to build predicted molecular functional network map of canonical pathways and associated genes with FOXM1 [13]. Survival analysis for FOXM1 expression vs patient survival in GBM patient samples was analyzed by curating publically available datasets derived from REpository for Molecular BRAin Neoplasia DaTa (REMBRANDT) (http://rembrandt.nci.nih.gov), for gliomas. For breast and lung cancer patient's univariate survival analysis by Kaplan- Meier curves was carried out using online KM-Plotter (www.kmplot.com) [21, 22]. The Km plotter Gene expression data and survival information contains data from GEO (Affymetrix HGU133A and HGU133+2 microarrays), EGA and TCGA).

### Statistical analysis

Data presented are the mean ± the standard deviation from three independent experiments unless indicated otherwise. All statistical tests were two-sided. For comparisons between groups, a Student's t test was used. Differences were considered to be statistically significant when p-value< 0.05.

## SUPPLEMENTARY MATERIALS METHODS AND FIGURES


