# Supplementary material for: FOXM1 and STAT3 interaction confers radioresistance in glioblastoma cells
Source: Oncotarget. 2016 Oct 14;7(47):77365–77. doi: 10.18632/oncotarget.12670 (PMC5340228; doi:10.18632/oncotarget.12670)
Supplement: Supplementary file 1 [file oncotarget-07-77365-s001.pdf]

FOXM1 and STAT3 interaction confers radioresistance in glioblastoma cells

SUPPLEMENTARY MATERIALS AND METHODS

Co-immunoprecipitation assay

U251 cells were treated with 4Gy RT for 24 hrs and cell lysates were prepared in RIPA lysis buffer (9.1 mM NaH 2PO4, 1.7 mM Na2HPO4, 150 mM NaCl, pH 7.4, 0.5% sodium deoxycholate, 1% Nonidet P-40, and 0.1% SDS, protease inhibitor cocktail). 100 µg of total protein was incubated with STAT3 or FOXM1antibody overnight at 4°C under constant rotation and 40ul protein A/G-agarose beads (#sc-2003, Santa Cruz., TX) were added to the mixture and incubated for 2-3 hrs at 4°C. The entire reaction mixture was gently washed three times with PBS with 0.1% tween-20 to remove the unbound proteins and the beads were resuspended in Laemmli solution and boiled for 5 min, resolved by SDS-PAGE, transferred to nitrocellulose membranes and probed overnight at 4°C with antibodies directed against FOXM1 or STAT3 and developed with chemiluminiscent reagent (Thermo Scientific., IL).

PIPs database; evidence of interaction between STAT3 and FOXM1

PIPs is a database (http://www.compbio.dundee.ac.uk/www-pips) of predicted human protein-protein interactions [20]. The predictions have been made using a naïve Bayesian classifier to calculate a Score of interaction. It contains predictions of >37,000 high probability interactions of which >34,000 are not reported in the interaction databases HPRD, BIND, DIP or OPHID. The probability of interaction between two proteins is calculated by combining different features including: expression, orthology, domain co-occurrence, post-translational modifications and sub-cellular location. The predictions also take account of the topology of the predicted interaction network. Score ≥1 indicating that the interaction is more likely to occur than not to occur. We compared interaction score between FOXM1 and STAT family members.

Homologous recombination (HR) repair assay using DR-GFP plasmid

This assay was performed as described elsewhere [46]. U251 cells were stably transfected with Lipofectamine as recommended by the manufacturer (Invitrogen) with 2 ug of circular pDR-GFP (Plasmid 26475., AddGene., USA), stable Puromycin-resistant colonies were selected with 2 to 6 µg/mL puromycin (cat: ant-pr-1., InvivoGen., USA). Selected U251-DR-GFP cells were maintained in DMEM medium supplemented with 10% fetal bovine serum (Invitrogen). The recombination assay relies on the two inactivated tandem repeated (DR)-GFP plasmid developed by M. Jasin [47].

Treatments

U251-DR-GFP cells (0.5 million in 60mm dishes) were treated with siomycin-A, 4 hours post transfection with 2ug I-SceI plasmid (pCBASceI) or mock. For siRNA mediated FOXM1 knock down, U251-DR-GFP cells were transfected with siRNA 24 hours prior to I-SceI transfection. Analysis was carried out 48 hours after I-SceI +/- transfection.

GFP analysis

GFP flow cytometry was performed on live cells by Fluroscence Flow cytometer (BD FACS caliber). Flow data was analysed using Flowing Software (http://www.flowingsoftware.com/).

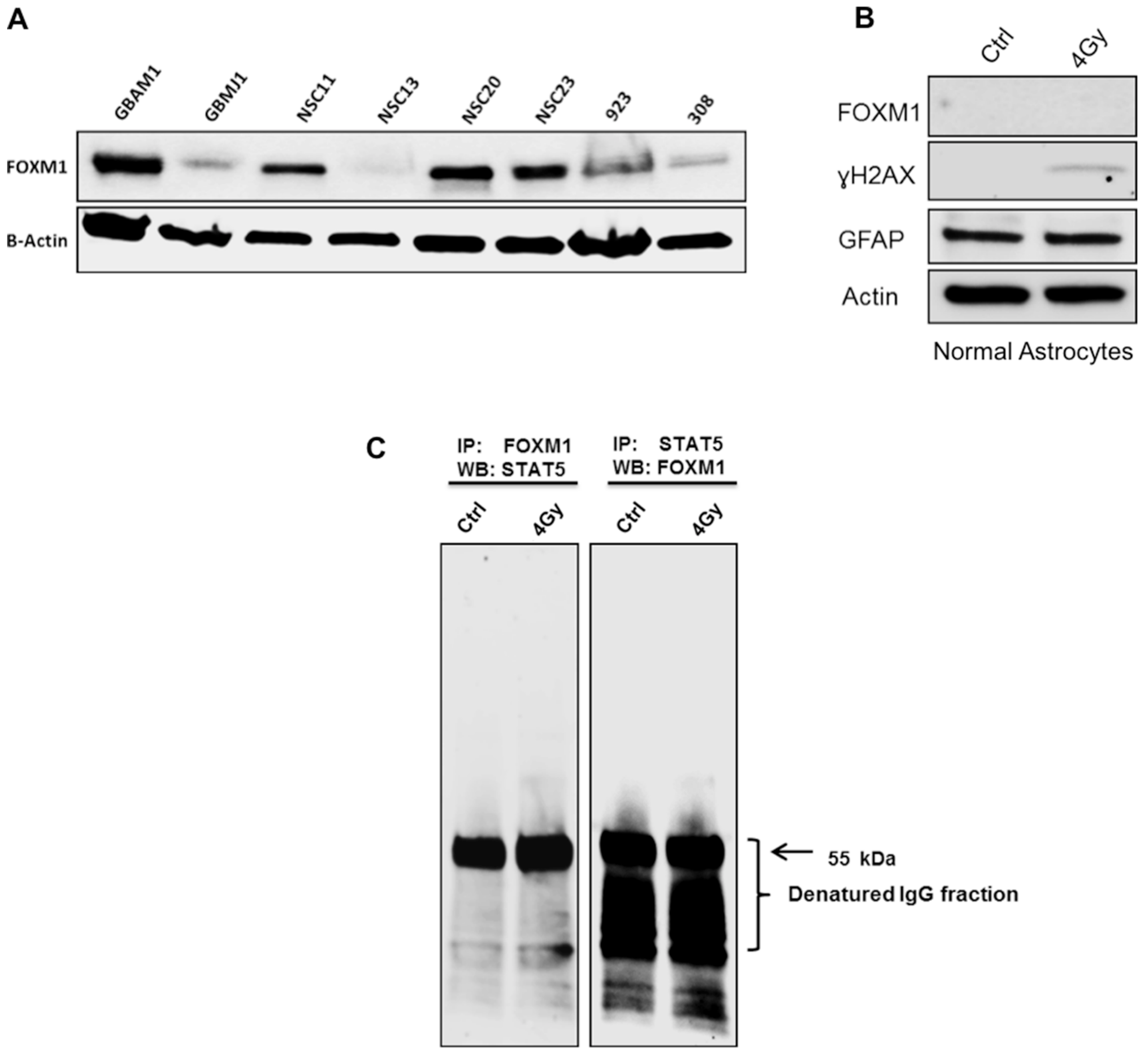

Supplementary Figure S1: FOXM1 expression in patient derived GBM stem cells and normal astrocytes. Panel **A.** represents immunoblot for FOXM1 in various patient derived GBM stem cells and panel **B.** represent immunoblot for FOXM1 in cultured normal astrocytes. Panel **C.** represent the co-immunoprecipitation immunoblots for FOXM1 and STAT5 interaction. Data presented are representative from at least three independent experiments.

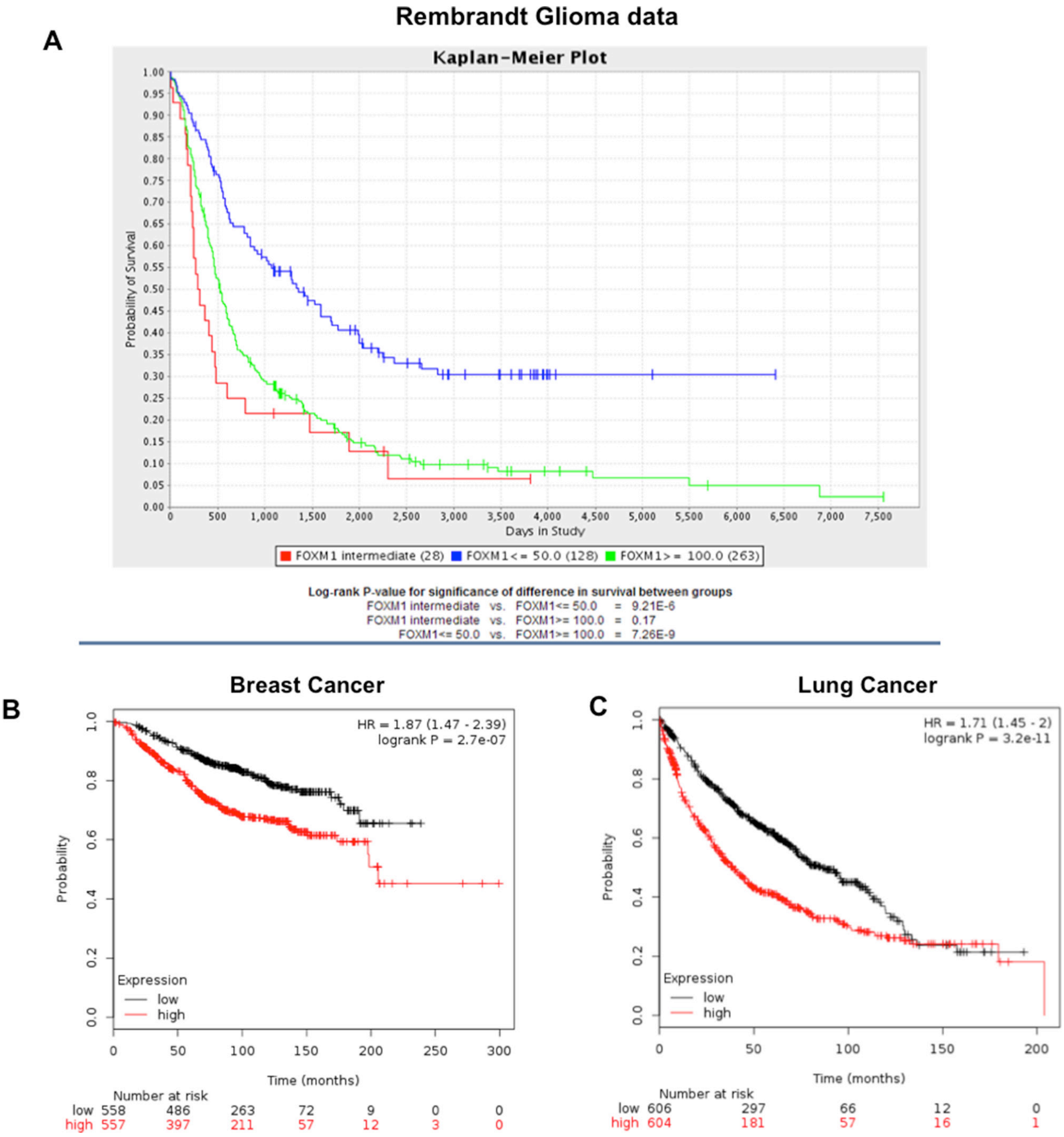

Supplementary Figure S2: Low levels of FOXM1 confers survival advantage in GBM, Breast and Lung cancer patients **Panel A.** FOXM1 expression levels were correlated to GBM patient survival as Kaplan-Meier plot by curating publically available datasets derived from REpository for Molecular BRAin Neoplasia DaTa (REMBRANDT) (http://rembrandt.nci.nih.gov), for all gliomas. The green line shows the survival of all glioma patients with intermediary FOXM1 expression. The red line shows the survival of FOXM1 over-expressing gliomas. While the Blue symbol show the patients that survived with a low expression of FOXM1. Kaplan-Meier plot survival analysis was carried out on data from individuals with breast panel **B.** or lung cancer panel **C.** using KM-Plotter (www.kmplot.com). This tool uses data from Gene Expression Omnibus (GEO, Affymetrix HGU133A and HGU133+2 microarrays), The European Genome-phenome Archive (EGA) and TCGA.

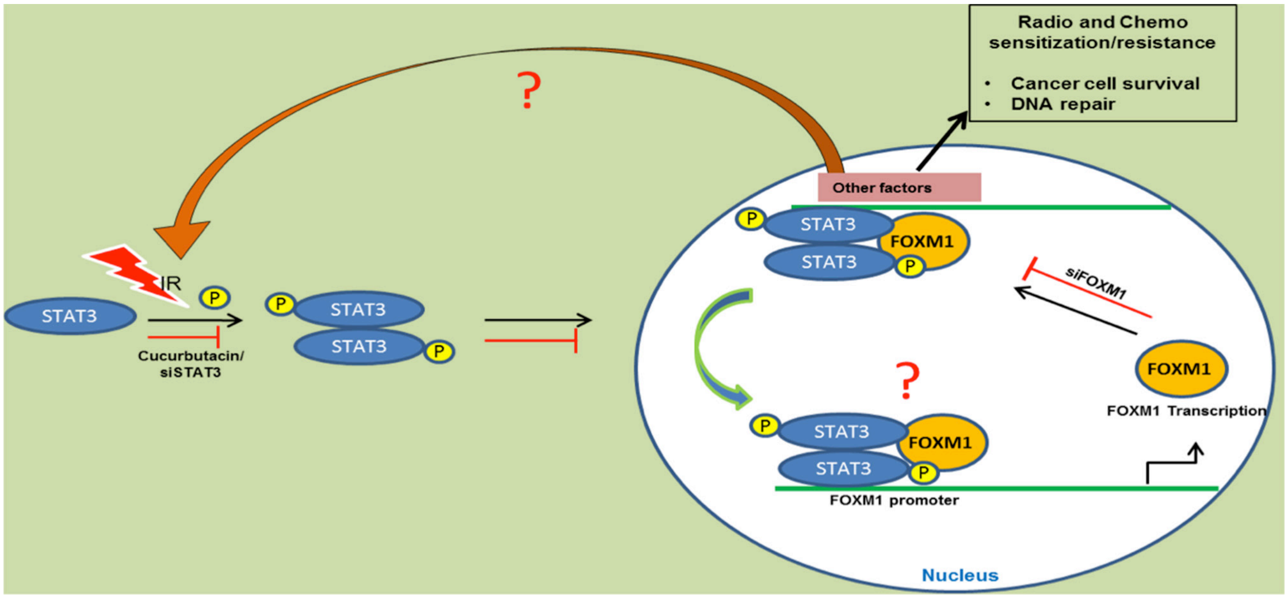

Supplementary Figure S3: Illustrative image. Predicted co-regulatory positive feedback loop mechanism between FOXM1 expression and STAT3 activation. We hypothesize FOXM1 and STAT3 interact and co-regulate their expression(FOXM1) and activation (pSTAT3). Further this interaction (FOXM1/pSTAT3) might regulate radio resistance in GBM cells.
